# Gaps in Community-Based Screening for Non-Communicable Diseases in Saudi Arabia

**DOI:** 10.3390/diseases13120407

**Published:** 2025-12-18

**Authors:** Ghadeer Al Ghareeb, Zaenab M. Alkhair, Zainab Alradwan, Hussain Alqaissoom, Horiah Ali Soumel, Khadijah R. Alsaffar, Fatema Muhaimeed, Burair Alsaihati, Mohammad N. Alkhrayef, Ibrahim Alradwan

**Affiliations:** 1 Qatif Primary Healthcare Center, Eastern Health Cluster, Ministry of Health, Al Qatif 32654, Saudi Arabia; gal-ghareeb@moh.gov.sa (G.A.G.); zalkhair@moh.gov.sa (Z.M.A.); zaalradwan@moh.gov.sa (Z.A.); halqaissoom@moh.gov.sa (H.A.); hsoumel@moh.gov.sa (H.A.S.); kalsaffar@moh.gov.sa (K.R.A.); 2 Radiation Oncology, Johns Hopkins Aramco Hospital, Dhahran 31311, Saudi Arabia; fatema.muhaimeed@jhah.com; 3 Applied Genomic Technologies Institute, King Abdulaziz City for Science and Technology (KACST), P.O. Box 6086, Riyadh 11442, Saudi Arabia; balsaihati@kacst.gov.sa; 4 Disability Research Institute, Health Sector, King Abdulaziz City for Science and Technology (KACST), Riyadh 11442, Saudi Arabia; mkhuryef@kacst.gov.sa; 5 Advanced Diagnostics and Therapeutics Institute, King Abdulaziz City for Science and Technology (KACST), P.O. Box 6086, Riyadh 11442, Saudi Arabia

**Keywords:** non-communicable diseases, community-based screening, preventive health, health promotion campaigns

## Abstract

Background: Non-communicable diseases (NCDs) such as cardiovascular diseases, diabetes, obesity, and cancer are the leading cause of mortality globally and in Saudi Arabia, accounting for more than 70% of all deaths. Despite national initiatives offering free preventive services, screening uptake remains low. This study aimed to describe the demographic and clinical characteristics of individuals participating in community-based NCD screening campaigns in the Eastern Province of Saudi Arabia and to evaluate screening uptake, compliance, and diagnostic outcomes. Methods: A retrospective cross-sectional analysis was conducted among 3106 adults screened at volunteer-driven community campaigns held between January 2023 and December 2024. Screening included anthropometric measurements, blood pressure assessment, and glucose testing, followed by eligibility evaluation for osteoporosis and cancer screening. Uptake and compliance were verified using electronic health records. Descriptive and inferential statistical analyses were applied. Results: Participants were 64% male and 36% female, with a mean age of 41.4 ± SD years. Obesity, hypertension, and diabetes were identified in 32%, 31%, and 12% of participants overall. Gender-stratified prevalence showed higher obesity among females at 36% (95% CI 32.3 to 38.1) and higher hypertension and diabetes among males at 36% (95% CI 34.0 to 38.2) and 14% (95% CI 12.1 to 15.2), respectively. Uptake among eligible individuals was 51% for dual-energy X-ray absorptiometry (DEXA), 47% for fecal immunochemical testing (FIT), 43% for Pap smear, and 39% for mammography. Diagnostic findings demonstrated substantial undetected disease burden, including osteoporosis in 41% (95% CI 26.0 to 56.8) of DEXA scans, a FIT positivity rate of 5% (95% CI 1.5 to 10.3), abnormal Pap cytology in 3% (95% CI 1.1 to 7.5), and BI-RADS 0 mammograms in 19% (95% CI 11.9 to 29.5), reflecting incomplete assessments requiring further evaluation. Conclusions: Community-based campaigns can effectively resolve limited engagement in health promotional activities and detect substantial burdens of undiagnosed NCDs. However, improvements in referral tracking, follow-up systems, and culturally tailored health education are essential to enhance screening compliance and early detection outcomes. These results can be utilized to inform public policies by extending screening services to additional areas, increasing investment in preventive health campaigns, and enhancing the capacity of the health system.

## 1. Introduction

Non-communicable diseases (NCDs), including cardiovascular diseases (CVD), diabetes mellitus, cancer, and chronic respiratory illnesses, have emerged as the predominant cause of global morbidity and mortality, accounting for 73% of all deaths worldwide. The burden is particularly acute in low- and middle-income countries (LMICs), where 80% of these deaths occur, placing immense pressure on healthcare systems and national economies [1,2]. Saudi Arabia mirrors this global trend, with 73% of all deaths attributable to NCDs, an alarming figure that emphasizes the urgency of implementing scalable and evidence-based preventive strategies [3,4].

In the Gulf Cooperation Council (GCC) region, and particularly in Saudi Arabia, the escalating burden of NCDs is closely linked to modifiable lifestyle factors, including physical inactivity, unhealthy dietary habits, obesity, and tobacco use [5]. According to the Saudi Arabia NCDs Report 2022, over 59% of adults in the Kingdom are classified as overweight or obese, with obesity alone affecting 24.7% of the population [5]. These risk factors significantly contribute to the high prevalence of CVD, type 2 diabetes, and several cancers, which collectively account for a substantial proportion of morbidity and mortality in the country [6]. Furthermore, dietary risks, such as the consumption of high-fat, high-sugar, and processed foods, exacerbate metabolic disorders and further elevate the NCD burden [7,8,9], as direct healthcare spending attributable to obesity in Saudi Arabia is estimated at SAR 14.4 billion (USD 3.8 billion), representing 4.3% of total national health expenditure [10]. When indirect costs such as lost productivity and disability are considered, the overall economic burden of NCDs rises to SAR 76 billion (USD 20.3 billion) annually, equivalent to approximately 3% of GDP [10]. Modeling studies suggest that cost-effective measures, including salt reduction, tobacco control, and improved primary care for CVD and diabetes could yield a return on investment of USD 7.3 per USD 1 spent, exceeding the global average of USD 4.0 [11]. Implementing these interventions could also prevent 162,000 premature deaths and gain over one million healthy life-years within 15 years, according to the national health strategy [12].

Saudi Arabia is committed to reducing premature NCD-related mortality by one-third in alignment with the WHO Global NCDs Action Plan and the Sustainable Development Goals (SDG Target 3.4) [13], and emphasizes prevention, early detection, and community-level awareness as central components of the health system reform [14].

Despite the availability of preventive screening services, including assessments for diabetes, hypertension, obesity, osteoporosis, and the most common cancers (breast, colorectal, and cervical), uptake remains consistently low across the Kingdom [15,16]. National data have shown suboptimal participation in routine checkups and preventive screenings, with cultural attitudes, limited health literacy, and reactive rather than preventive health-seeking behaviors among Saudis identified as contributing factors [17]. For example, 92% of women aged 40^+^ had never received a mammogram, and 89% lacked a clinical breast exam, despite the availability of free services [17,18]. In most cases, individuals seek medical attention in response to acute or severe symptoms, bypassing the preventive measures [19].

These behaviors contribute to delayed diagnoses, more advanced disease at presentation, poorer outcomes, and higher healthcare costs [20,21]. To address this, Saudi health authorities have expanded community-based NCD screening campaigns to improve awareness, correct misconceptions, and provide accessible pathways for early detection and intervention, therefore reducing complications and mortality. Evidence suggests that such initiatives can enhance health-seeking behavior and identify individuals at high risk who would otherwise remain undiagnosed [22]. However, these community-based NCD screening campaigns remain under-evaluated in the country.

In Saudi Arabia, limited data exist on participant characteristics, screening uptake, and subsequent compliance with follow-up interventions in national health campaigns. This gap hinders the ability of health policy makers and program implementers to refine interventions, allocate resources effectively, and potentially exacerbate health disparities [23]. Additionally, this gap makes it difficult to assess the effectiveness of existing community outreach methods and establish best practices. Evaluating the outcome of the community-based NCD screening campaigns may help in creating sustainable health improvement strategies within the community.

This study examines the demographic and clinical profiles of individuals attending a large community-based NCD screening campaign in the Eastern Province. It evaluates eligibility, screening uptake, and compliance across major domains, including metabolic risk, osteoporosis, and common cancers. It also introduces three key methodological strengths: a multimodal assessment of screening behaviors, verification of completed referrals through linked electronic records, and population-normalized estimates of campaign reach. Together, these features provide a more accurate and policy-relevant evaluation of community-based screening performance in Saudi Arabia.

## 2. Materials and Methods

### 2.1. Study Design

This investigation was a cross-sectional, observational study, conducted with the aims of describing population characteristics and evaluating the existing regional NCD awareness and screening community-based campaign conducted between January 2023 and December 2024 in the Eastern Province of Saudi Arabia. The campaigns were organized in high-traffic public areas, including shopping malls, seashores, and various governmental and private institutions. These settings were chosen to enhance community engagement and accessibility for a limited engaged population in health promotional activities. Participant identifiers collected during the screening campaign were cross-matched with patient records retrieved from Al Qatif Primary Health Care centers’ electronic medical records. This allowed for the validation of test uptake and diagnostic results over the 24-month follow-up period after referral.

### 2.2. Ethical Considerations

The study protocol was reviewed and approved by the Institutional Review Board of Qatif Central Hospital (Approval No. QCH-SRECO 61/2023). All participants provided informed verbal consent before participation. The study adhered to the principles outlined in the Declaration of Helsinki for research involving human subjects.

### 2.3. Sample Size Calculation

The minimum required sample size was estimated using OpenEpi Version 3.01 (SSPropor function) to achieve 80% statistical power at a 5% significance level (two-tailed). The proportion of 22.9% for periodic wellness visits was selected as a conservative proxy for baseline engagement with preventive health services [4]. This ensured adequate estimate of screening uptake and compliance. Using this estimate, with a design effect of 1.0 (simple random sampling) and an anticipated 20% rate of nonresponse or missing data, the minimum required sample size was 327 participants. The final analytic sample of 3106 unique participants substantially exceeded this threshold, providing high precision for all reported estimates. This higher sample number reflects strong community engagement and broad campaign reach, supported by enthusiastic participant response and external institutional backing.

### 2.4. Study Population

The target population included adults aged 18 years and older residing in the Eastern Province. A total of 3455 individuals were initially enrolled. Repeat participants were identified through matching of national identification numbers across all campaign events, and individuals who appeared more than once were excluded to ensure that the final dataset represented unique participants only. After applying this criterion and excluding cases with incomplete or missing data, 3106 participants were included in the final analysis. NCDs screened during the campaign included obesity, hypertension, diabetes mellitus, osteoporosis, and common cancers (breast, colorectal, and cervical).

Participants were invited by healthcare workers stationed at campaign venues. Upon consent, trained medical staff, using standardized protocols, collected anthropometric and clinical measurements, including height, weight, body mass index (BMI), systolic and diastolic blood pressure, random or fasting blood glucose levels, and smoking history. For participants eligible and willing to undergo further screening (DEXA, FIT, mammogram, Papanicolaou (Pap smear), follow-up appointments were scheduled at affiliated health centers. Screening uptake and results were tracked via electronic medical records for up to nine months post-enrollment. To assess actual compliance, participant national identification (ID) numbers were used as identifiers to be cross-matched with pooled electronic medical records from Qatif primary health care centers or the surveillance system implemented on web-based software “Research Electronic Data Capture (REDCap), version 14.3.10”, comprising 19,465 entries spanning all relevant screening tests conducted between January 2023 and December 2024. This linkage allowed verification of completed procedures following referral from the campaign.

### 2.5. Referral and Follow-Up Procedures

In accordance with the guidelines of the Centers for Disease Control and Prevention (CDC) and the Saudi Ministry of Health (MOH), participants with abnormal screening results were referred to appropriate healthcare facilities for follow-up evaluation and management.

For diabetes screening, individuals with a fasting blood glucose level of ≥126 mg/dL or a random/postprandial glucose level of ≥200 mg/dL were referred to their nearest primary healthcare center (PHC) for confirmation and further assessment. Participants presenting with severe hyperglycemia (>450 mg/dL) or marked hypoglycemia (<55 mg/dL; 3.0 mmol/L) unresponsive to oral carbohydrate intake, or those who were unconscious, unable to swallow, or experiencing seizures or severe dizziness, were immediately referred to the emergency department for urgent medical care.

For hypertension, referral to the PHC was made when elevated blood pressure was confirmed in two or more readings ≥140/90 mmHg following proper measurement and rest. Participants with severely elevated (≥180/110 mmHg) or hypotensive (<95/60 mmHg) readings accompanied by symptoms such as dizziness, chest pain, or shortness of breath were referred to the emergency department.

Participants meeting eligibility criteria for other preventive screenings were referred according to age group and screening type, consistent with national preventive guidelines. Those requiring bone mineral density assessment (DEXA scan) were directed to the mobile screening unit. In contrast, mammography for women aged 40–69 years was performed at Sayyid Ali Al-Salman Center or Qatif Central Hospital. Eligible individuals were also referred to PHCs for additional preventive screenings, including FIT for adults aged 45–75 years and Pap smear testing for married women aged 21 years and above, in line with approved national standards for early detection and health promotion.

### 2.6. Eligibility Criteria for Screening

**Figure 1:**
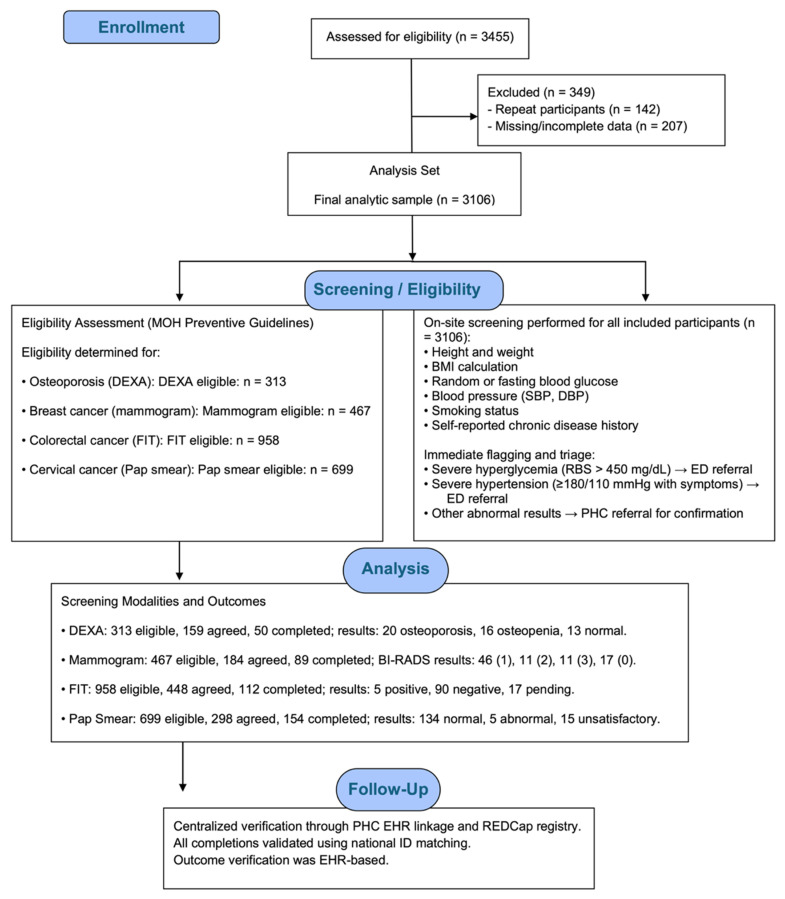
Participant flow diagram for the community-based NCD screening campaign, showing eligibility assessment, exclusion criteria, screening workflow, allocation to screening modalities (DEXA, mammography, FIT, and Pap smear), completion rates, and EHR-verified outcomes.

Eligibility was based on Saudi Ministry of Health guidelines and the “Know Your Numbers” initiative. All individuals aged ≥18 years were eligible for diabetes, hypertension, and obesity screening. Osteoporosis screening with DEXA was offered to postmenopausal women aged 60 years or older and to men aged 65 years or older. Cancer screenings were conducted according to national preventive guidelines: mammography for women aged 40–69 years at average risk, FIT for adults aged 45–75 years at average risk, and Pap smear for sexually active women aged 21–65 years. Figure 1 illustrates the participant flow throughout the community-based NCD screening campaign, detailing eligibility assessment, exclusions, on-site screening procedures, allocation to each screening modality, completion of diagnostic tests, and verification of outcomes through the electronic health record.

### 2.7. Data Collection

The dataset encompassed a comprehensive range of variables relevant to the assessment of NCD risk. Sociodemographic data included participants’ age and gender. Medical history was based on self-reported diagnoses of chronic conditions such as diabetes mellitus, hypertension, and other comorbidities, along with smoking status. Clinical and physical measurements were systematically recorded during the campaign, including height, weight, body mass index (BMI), Systolic blood pressure (SBP), Diastolic blood pressure (DBP), and random or fasting blood glucose (RBS or FBS) levels. In addition, screening eligibility and willingness to undergo further diagnostic tests, such as DEXA, FIT, mammography, and cervical cancer screening using the Pap smear, were documented as part of the follow-up process.

### 2.8. Operational Definitions

BMI was categorized according to the WHO classification: normal (18.5–24.9 kg/m^2^), overweight (25–29.9 kg/m^2^), and obese (≥30 kg/m^2^) [24]. Hypertension and diabetes were defined based on either a prior medical diagnosis, on medical treatment, or measurements exceeding standard clinical thresholds, e.g., SBP ≥ 140 mmHg, DBP ≥ 90 mmHg, Fasting blood sugar (FBS) ≥ 126 mg/dL, or Random Blood Sugar (RBS) ≥ 200 mg/dL [25]. Smoking status was categorized as current smoker (≥1 cigarette/day), ex-smoker (abstinent >1 year), or non-smoker [26]. Uptake rate was defined as the percentage of participants who were eligible and agreed to perform the test. The compliance rate was defined as the percentage of participants who agreed to perform the test and then did so at the health institute.

### 2.9. Statistical Analyses

Statistical analyses were performed using GraphPad Prism 8 (GraphPad Software, version 8, San Diego, CA, USA). Continuous variables were assessed for normality using visual inspection (histograms and violin plots) and the Shapiro–Wilk test. Variables demonstrating non-normal distributions were summarized and compared using non-parametric tests (Mann–Whitney U test). Normally distributed variables were presented as mean ± standard deviation (SD) and compared using independent-sample *t*-tests. Categorical variables were summarized as frequencies and percentages and compared using chi-square or Fisher’s exact tests. Gender-stratified analyses were performed for obesity, hypertension, diabetes, smoking status, and screening eligibility. The chi-square test was used when all expected cell frequencies were ≥5; Fisher’s exact test was applied when any expected count was <5, including small or unevenly distributed subgroups such as Pap smear abnormalities and FIT-positive results, to ensure valid probability estimation. Population normalization was conducted using demographic data from the Eastern Province obtained from the 2022 Saudi Census published by the General Authority for Statistics (GASTAT), which reported a regional population of 1,210,992 residents. Normalized rates were calculated using the equation: Normalized rate = (number of participants or cases ÷ total population) × 100,000. Two normalization frameworks were applied: (1) per 1000 screened participants to describe the internal yield of the campaign, and (2) per 100,000 residents to describe the population-level reach within the Eastern Province. All statistical tests were two-sided, and a *p*-value < 0.05 was considered statistically significant.

## 3. Results

### 3.1. Demographic and Risk Factor Distribution

A total of 3106 adults participated in the community-based NCD screening campaign, including 1972 males (64%) and 1134 females (36%). Males were significantly older than females (41.4 ± 12.9 vs. 38.8 ± 13.7 years, *p* < 0.001).

**Figure 2:**
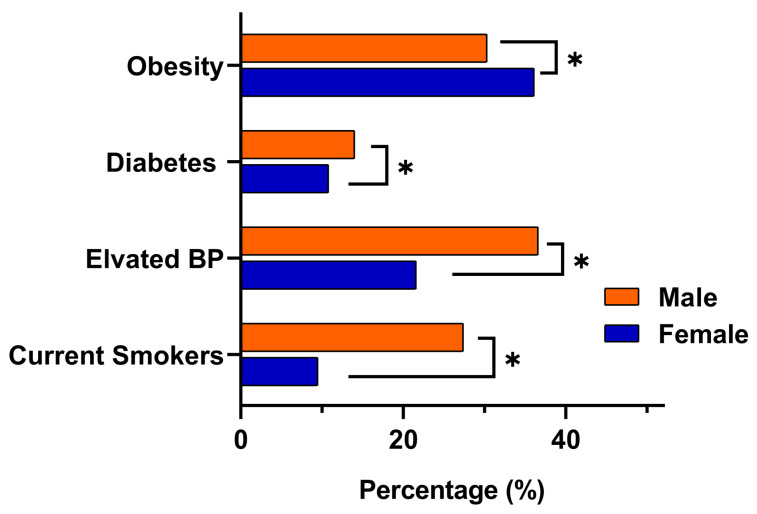
Gender-based comparison of key NCDs indicators among adults aged ≥18 years in eastern Saudi Arabia. Bars represent the proportion of male and female participants meeting clinical thresholds for obesity (BMI ≥ 30), elevated blood pressure (SBP ≥ 140 mmHg or DBP ≥ 90 mmHg), diabetes (self-reported or RBS ≥ 200 mg/dL or FBS ≥ 126 mg/dL), and current smoking. Asterisks (*) denote statistically significant differences between genders (*p* < 0.05).

Risk factor patterns differed markedly by gender. Obesity was more common among females, with a prevalence of 36% (95% CI 32.3 to 38.1), compared with 29% in males (95% CI 27.7 to 31.8). In contrast, males demonstrated significantly higher rates of elevated blood pressure at 36% (95% CI 34.0 to 38.2) versus 21% in females (95% CI 18.8 to 24.0) (*p* < 0.001), as well as higher diabetes prevalence at 14% (95% CI 12.1 to 15.2) compared with 10% in females (95% CI 8.7 to 12.4) (*p* < 0.009). Current smoking also showed a clear male predominance, affecting 27% of males (95% CI 25.0 to 28.9) compared with 9% of females (95% CI 7.4 to 10.8) (*p* < 0.001). Consistent with these metabolic and behavioral differences, eligibility for colorectal cancer screening using FIT was higher in males (33%) than females (27%). A visual comparison of the key NCD indicators obesity, elevated blood pressure, diabetes, and smoking is presented in Figure 2.

### 3.2. Uptake and Compliance of Preventive Screening

**Figure 3:**
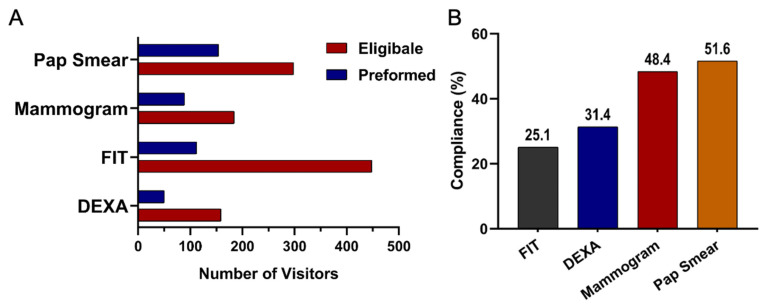
(**A**) Number of individuals eligible and those who underwent screening for the non-communicable disease tests: Mammogram, FIT, DEXA, and Pap Smears. (**B**) Compliance rates across screening test types: FIT (Fecal Immunochemical Test), DEXA (Dual-Energy X-ray Absorptiometry), and Mammogram.

Among eligible participants, willingness to undergo screening varied across test types. Uptake was highest for DEXA at 51% (95% CI 45.1 to 56.0), followed by FIT at 47% (95% CI 43.7 to 49.9), Pap smear at 43% (95% CI 38.6 to 46.5), and mammography at 39% (95% CI 34.7 to 44.3). Compliance patterns, however, differed from uptake. Pap smear screening demonstrated the highest compliance at 51% (95% CI 45.9 to 57.2), followed by mammography at 48% (95% CI 41.0 to 55.8). Compliance was lower for DEXA at 31% (95% CI 24.6 to 39.1) and lowest for FIT at 25% (95% CI 21.2 to 28.7). Taken together, these results highlight substantial variation in both willingness to participate in screening and follow through after initial agreement. These uptake and compliance patterns are summarized in Table 1 and visualized in Figure 3. A cascading dropout pattern was evident across all screening steps. Although many participants were eligible, uptake declined at the consent stage for all modalities, and an additional drop occurred between uptake and completion. Completion was lowest for DEXA (31%) and FIT (25%) compared with higher completion among those who agreed to mammography (48%) and Pap smear screening (51%). This progressive loss across the screening pathway highlights key barriers to successful follow through.

### 3.3. Tests Performed Among Eligible Participants

The number of individuals eligible for screening compared to those who ultimately completed the test across the major modalities of mammography, FIT, DEXA, and Pap Smear is presented in Table 1 and Figure 3A. Among the tests, Pap smear screening showed the highest completion rate among eligible participants (22%). Mammography followed with a moderate uptake among eligible participants (19%). DEXA had a low completion rate (16%). In contrast, FIT, despite having the largest pool of eligible individuals, showed the lowest follow-through rate (112 out of 958 participants, 12%).

### 3.4. Screening Outcomes

The diagnostic outcomes from follow-up screenings revealed a considerable burden of previously undetected NCD-related conditions. Among women undergoing mammography (*n* = 89), 52% had normal findings (BI-RADS 1), 17% had benign findings (BI-RADS 2), and 12% were classified as BI-RADS 3, which require short-interval follow-up; the proportion classified as BI-RADS 3 corresponded to 12% (95% CI 10.4 to 20.8). Additionally, 19% of women received BI-RADS 0 results (95% CI 11.9 to 29.5), indicating incomplete assessment and the need for further imaging. For colorectal cancer screening, 112 individuals completed FIT, of whom 5% tested positive (95% CI 1.5 to 10.3), while 80% tested negative and 15% had pending or undocumented results. Among the 49 participants who underwent DEXA scanning, osteoporosis was detected in 41% (95% CI 26.0 to 56.8), osteopenia in 32% (95% CI 20.5 to 46.0), and normal bone density in 27% (95% CI 15.4 to 40.2). Cervical cancer screening showed that 87% of Pap smears demonstrated normal cytology, while abnormal cervical changes were identified in 3% of women (95% CI 1.1 to 7.5), and 10% of samples were rejected due to inadequate specimen quality. Detailed results are presented in Table 2.

### 3.5. Gender-Specific Patterns

**Figure 4:**
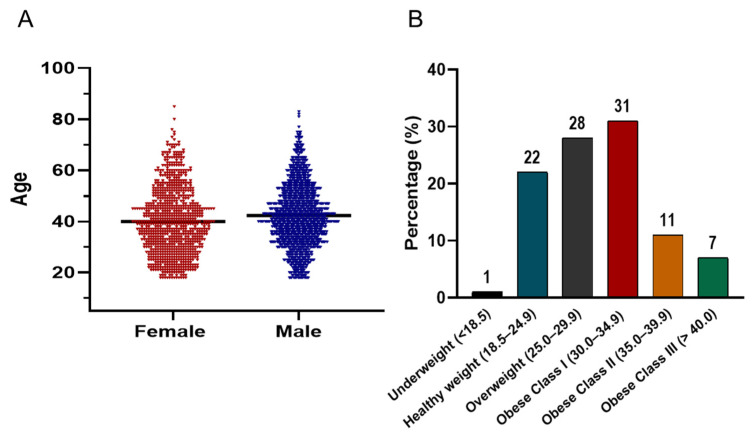
(**A**) Violin plots comparing the age distribution of male (blue) and female (red) participants in the screening campaign. The plots display kernel density estimates to visualize the spread and central tendency of ages, with the majority of both groups concentrated between 30–50 years. The black horizontal line within each violin indicates the median value. (**B**) Bar chart showing the distribution of BMI categories among all participants.

Figure 4A illustrates the age distribution of the screened cohort, which follows a bell-shaped histogram pattern. The majority of participants were concentrated between the ages of 30 and 50 years. In terms of BMI distribution (Figure 4B), the prevalence of being overweight and obese was striking. Only 1% of the population was underweight, while 22% had a healthy weight (BMI 18.5–24.9). In contrast, 28% were classified as overweight (BMI 25–29.9), and 49% met criteria for obesity. Among those with obesity, 31% were categorized as Class I (BMI 30–34.9), 11% as Class II (BMI 35–39.9), and 7% as Class III (BMI ≥ 40).

**Figure 5:**
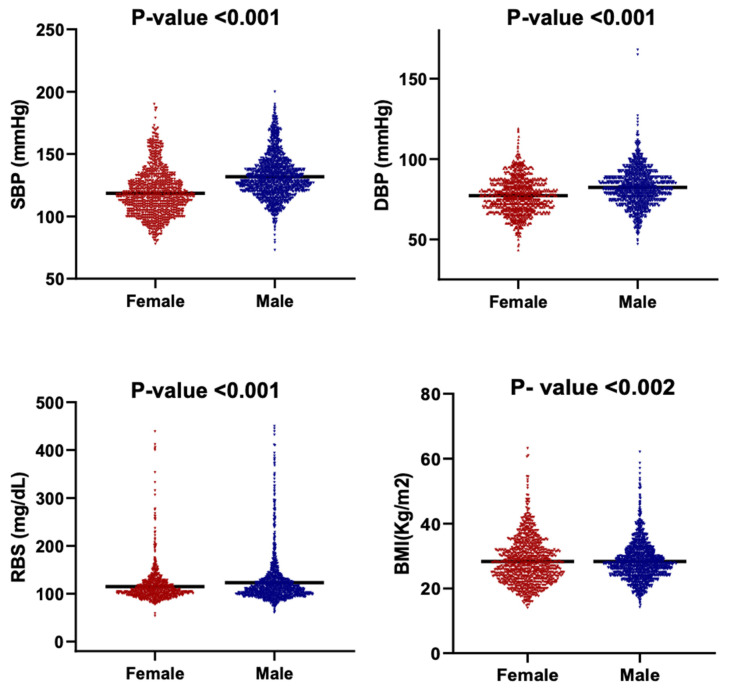
Violin plots showing the distribution of key clinical parameters by gender. The parameters are SBP, DBP, RBS and BMI. Each subplot compares the distribution between females and males. The black horizontal line within each violin indicates the median value.

Figure 5 presents the distribution of BMI, SBP, DBP, and RBS among male and female participants using violin plots that depict both data density and the full range of observed values. Median-based comparisons were employed due to the non-normal distributions and unequal variances evident across multiple parameters.

There is a clear sex-based difference observed in cardiometabolic indicators. Males demonstrated significantly higher blood pressure values, with a median SBP of 129 mmHg compared with 116 mmHg in females, and a median DBP of 82 mmHg versus 77 mmHg in females (*p* < 0.001). Median RBS followed the same pattern, with males showing higher glycemic levels (110 mg/dL) relative to females (105 mg/dL) (*p* < 0.001). The broader distributions of SBP and RBS among males further indicate greater inter-individual variability in blood pressure and glucose regulation.

The BMI distributions differed in shape between males and females (Kolmogorov–Smirnov *p* < 0.002), although median BMI values did not differ significantly as the Mann–Whitney test had a *p* > 0.05. This indicates that sex-based variation in adiposity is driven primarily by distribution width rather than central tendency, with females exhibiting a more compact clustering around the median.

When normalized to the Eastern Province population of 1.21 million residents (GASTAT 2022), the campaign reached 257 participants per 100,000 individuals (Table 3). The normalized prevalence of hypertension and obesity was 79 and 81 per 100,000, respectively. Participation in cancer and bone health screening was lower, with rates ranging from 4 to 15 per 100,000 residents. Mammography demonstrated the highest participation at 15 per 100,000, followed by Pap smears at 13 per 100,000. These population-adjusted values provide a clearer estimate of the campaign’s reach and screening yield within its effective catchment area.

## 4. Discussion

This community-based screening campaign research in the Eastern Province of Saudi Arabia evaluated important gaps in preventive care regarding the effectiveness of the NCD screening campaign in the country in terms of participant characteristics, screening uptake, and compliance rate. The aim of community-based screening campaign is early detection and intervention, which lowers mortality and morbidity.

This study revealed a substantial burden of NCDs. The prevalence of obesity, hypertension, and diabetes was notable, with distinct gender disparities as obesity was more prevalent among females (36.0%), while hypertension, diabetes, and tobacco use were higher in males (36.6%, 14.0%, and 26.9%, respectively) [27,28]. This highlights the distinct gender-specific distribution of metabolic and behavioral risk factors. The present campaign detected a higher prevalence of Diabetes (12.4%) than national estimates (exceed 11%); the national projections indicate there could be an increase by more than 100% by 2050 [29,30].

These findings highlight the substantial underlying burden of NCDs identified through community screening. Furthermore, they highlight distinct and consistent gender-related differences in cardiometabolic profiles within the screened population and stress the need for NCD prevention and management efforts in community settings. Beyond clinical burden, the population and economic stakes are considerable. In Saudi Arabia, NCDs account for ~73% of deaths and drive substantial direct and indirect costs [31,32].

Total participants numbered 3106. At the population level, campaign participation corresponded to 257 per 100,000 adults in the Eastern Province. Screening-specific engagement ranged from 4.0 to 15 per 100,000, with the highest participation observed for mammography and Pap smear screening (Table 3) [33].

Most of the participants were between the ages of 30 and 50 years, indicating a predominance of middle-aged individuals in the population engaged with the screening campaign. This age group represents a critical window for the early detection and prevention of NCDs, further emphasizing the value of timely community-based screening interventions.

Despite broad eligibility, screening uptake across modalities remained below recommended benchmarks, WHO and national targets for effective population-level impact [34] [35,36]. Only 19% of eligible women underwent mammography, 16% completed DEXA scans, and 12% completed FIT testing, while Pap smear screening achieved the highest participation rate at 22%. For Pap smear testing, 3% of women showed abnormal cytology, while 15 samples (10%) were rejected due to inadequate quality.

Gender-specific eligibility patterns were also observed. The FIT eligibility was higher in men than in women consistent with global evidence linking colorectal cancer risk to smoking, diet, and healthcare-seeking behavior [37]. Eligibility for osteoporosis screening was similar between sexes (11% vs. 10%), highlighting the underdiagnosis of osteoporosis in men [38].

Barriers to screening uptake are complex and multifactorial, encompassing social, cultural, and systemic factors. Cultural stigma, misconceptions about screening procedures (such as fear of radiation exposure or cancer diagnosis), and limited health literacy remain prominent deterrents [29,39,40,41,42]. Physician recommendation also exerts a strong influence on participation; in Aljouf (another region in Saudi Arabia), 52.7% of women reported undergoing mammography only following a physician’s referral [40,43,44,45].

Evidence from Saudi Arabia and the wider Gulf further emphasizes these systemic barriers. In a national study, although over 4000 women expressed interest in osteoporosis screening, only 15% completed the test [46]. Similarly, in Jazan, 90% of women had never undergone DEXA, most citing lack of physician recommendation (46%) or awareness (44%) [47]. In contrast, UAE data showed that older adults demonstrated relatively higher awareness, with 50% of those aged over 55 reporting good practices toward osteoporosis screening [48]. These gender patterns also align with international data [45,49].

Our diagnostic outputs clarify where the system can improve. Among those who proceeded to DEXA, prioritizing bone-health pathways is important, including referral navigation in case tracking. For colorectal screening, documented drop-off and incomplete follow-up highlight gaps in referral navigation and tracking [34]. Mammography highlighted challenges such as BI-RADS 0 incomplete assessments, emphasizing the need for timely additional imaging and stronger recall systems. Meanwhile, Pap smear experiences similarly revealed issues with inadequate sampling and specimen rejection, indicating opportunities to reinforce technical quality assurance and targeted staff training.

Programmatically, three actions are likely to yield immediate gains. First, embedding screening within routine primary care through e-referrals and automated reminders may reduce attrition and increase the proportion of completed tests. Second, targeted, culturally tailored education is essential to convert early detection into scalable outcomes designed to address stigma, fear of diagnosis or radiation, and other misconceptions reported across Saudi and UAE studies [32,34,42]. Third, strengthening quality-assurance processes, including standardized sampling checklists, periodic staff refreshers, and rapid recall pathways for BI-RADS 0 findings, can improve the diagnostic yield of completed tests. These steps address both the structural and behavioral barriers in support of the preventive health goals initiatives outlined in Vision 2030.

This research is subject to several potential sources of bias. The cross-sectional design inherently limits the ability to account for confounding variables, and no statistical adjustments were performed to control these factors. Self-selection bias is also likely, as the findings reflect only individuals who voluntarily attended the community campaigns and may not be fully representative of the broader population. However, campaign invitations were distributed evenly across beneficiaries, which may have partially mitigated this limitation. Future campaigns and studies should integrate automated e-referrals, gender-sensitive education, and quality assurance systems to improve compliance and diagnostic accuracy.

## 5. Conclusions

In sum, our results demonstrate a high gender patterned burden of NCDs and meaningful screening outcomes from community outreach, yet they also reveal inconsistencies between eligibility, uptake, and follow through. Integrating screening into primary care, strengthening referral and recall systems, and providing culturally tailored education are essential to convert early detection into scalable and improved health outcomes. Additionally, our findings can be utilized to guide evidence-based policies by extending the comprehensive screening services to wider areas, increasing investment in preventive health campaigns, and enhancing the capacity of the health system to effectively provide the additional medical management interventions required.

## Figures and Tables

**Table 1:** Comparison of Eligibility, Uptake, Compliance, and Completion Rates for DEXA, FIT, Mammography, and Pap Smear Screening.

	Eligible (*n*)	Agreed (*n*)	Performed (*n*)	Uptake Rate (%)	Compliance Rate (%)	Performed/Eligible (%)
DEXA	313	159	50	51%	31%	16%
FIT	958	448	112	47%	25%	12%
Mammogram	467	184	89	39%	48%	19%
Pap Smear	699	298	154	43%	51%	22%

DEXA: Dual-energy X-ray absorptiometry, FIT: fecal immunochemical test, Pap smear: Papanicolaou smear.

**Table 2:** Detailed outcomes of breast cancer (mammogram), colorectal cancer (FIT), and osteoporosis (DEXA) screenings in patients referred for hospital follow-up from primary care campaigns in Eastern Saudi Arabia.

Screening Test	Total Tested (*n*)	Normal Findings N (%)	Borderline Findings N (%)	Abnormal Findings N (%)	Indeterminate/Missing N (%)
Mammogram	89	BI-RAD 1 46 (52%)	BI-RAD 2 15 (17%)	BI-RAD 3 11 (12%)	BI-RAD 0 17 (19%)
FIT	112	90 (80%)	N/A	FIT-positive 5 (5%)	Pending 17 (15%)
DEXA	49	13 (27%)	Osteopenia 16 (32%)	Osteoporosis 20 (41%)	N/A
Pap Smear	154	134 (87%)	N/A	Pap Smear-positive 5 (3%)	Rejected samples 15 (10%)

DEXA: Dual-energy X-ray absorptiometry, FIT: fecal immunochemical test, Pap smear: Papanicolaou smear, N/A: not applicable.

**Table 3:** Population-normalized rates of screening and clinical findings among participants of the community-based NCD campaign.

Parameter	Raw Count (3106)	Per 1000 Screened	Per 100,000
Hypertension (elevated BP)	951 (30.6%)	306	79
Obesity	983 (31.6%)	317	81
Current smokers	632 (20.3%)	203	52
DEXA completed	49 (1.6%)	16	4
FIT completed	112 (3.6%)	36	9
Mammogram completed	184 (5.9%)	60	15
Pap smear completed	154 (5.0%)	50	13

## Data Availability

The data supporting the findings of this study are not publicly available due to privacy and ethical restrictions, as they contain sensitive patient information from electronic health records at Qatif Central Hospital. Requests for access to anonymized data may be considered on a case-by-case basis by the Institutional Review Board of Qatif Central Hospital.

## Abbreviations

The following abbreviations are used in this manuscript:

Abbreviation	Full Term
BMI	Body Mass Index
BP	Blood Pressure
SBP	Systolic Blood Pressure
DBP	Diastolic Blood Pressure
RBS	Random Blood Sugar
FBS	Fasting Blood Sugar
DEXA	Dual-Energy X-ray Absorptiometry
FIT	Fecal Immunochemical Test
Pap smear	Papanicolaou Smear
NCDs	Non-Communicable Diseases
CVD	Cardiovascular Disease
GCC	Gulf Cooperation Council
LMICs	Low- and Middle-Income Countries
ROI	Return on Investment
MOH	Ministry of Health
KACST	King Abdulaziz City for Science and Technology
BI-RADS	Breast Imaging Reporting and Data System
IRB	Institutional Review Board
SD	Standard Deviation
